# Evaluation of efficacy of iodine prophylaxis in Poland based on the examination of schoolchildren living in Opoczno Town (Lodz Voivodship)

**DOI:** 10.1186/1756-6614-5-23

**Published:** 2012-12-22

**Authors:** Arkadiusz Zygmunt, Zbigniew Adamczewski, Katarzyna Wojciechowska-Durczyńska, Anna Cyniak-Magierska, Kinga Krawczyk-Rusiecka, Agnieszka Zygmunt, Małgorzata Karbownik-Lewińska, Andrzej Lewiński

**Affiliations:** 1 Polish Mother’s Memorial Hospital Research Institute, Lodz, Poland; 2 Department of Endocrinology and Metabolic Diseases, Medical University of Lodz, Lodz, Poland; 3 Department of Pediatric Cardiology and Rheumatology, Medical University of Lodz, Lodz, Poland; 4 Department of Oncological Endocrinology, Chair of Oncological Endocrinology, Medical University of Lodz, Lodz, Poland

**Keywords:** Iodine prophylaxis, Goitre, Schoolchildren, Urine iodine concentration

## Abstract

**Background:**

In 1997 a currently obligatory model of iodine prophylaxis, based on mandatory iodization of household salt with 30 mg KI/kg, was introduced. The aim of our study was to assess the iodine intake among school-age children living in Opoczno in 3 subsequent time points – in 1994, before establishment of currently operating model of iodine prophylaxis, in 1999 – 2 years after implementation of iodine prophylaxis and in 2010, – 14 years after its implementation.

**Methods:**

We assessed goitre incidence and urine iodine concentration (UIC) in 104 children in 1994, 207 children in 1999 and 174 children in 2012. Age of examined children ranged from 6 to 15 years. The thyroid volumes evaluated by ultrasound were compared to reference values for thyroid volume proposed by Zimmermann at al. Moreover, we have introduced a new index – V/BSA ratio (comparison of thyroid volume to the body surface area), which to our belief allows for more accurate assessment of thyroid volume.

**Results:**

The median of UICs was 45.5 μg/L (1994), 101.1 μg/L (1999) and 100.6 μg/L (2010). The distribution of obtained results has changed as well – iodine concentrations below 50 μg/L were present in 59.1% children in 1994, in 12.6% children – in 1999 and in 7.1% children – in 2010.

Although a significant decrease in goitre incidence with regard to age – 92.6% (1994) vs 18.5% (1999) and 15.8% (2010), as well as with regard to BSA – 95.4% (1994) vs 15.2% (1999) and 11.6% (2010) was observed, it still points to the iodine deficiency, which is in contradiction with UICs as they are within normal limits. V/BSA ratio avoids such discrepancy. The values of ratio V/BSA were higher in 1994 (7.079 ± 2.775) than in 1999 (2.935 ± 1.112) (p<0.05) and in 2010 (2.846 ± 1.029) (p<0.05).

**Conclusions:**

Hitherto model of iodine prophylaxis has proved to be effective in eliminating the iodine deficiency. The iodine intake is now more even, homogenous, which translates into smaller scatter of UICs and less percentage of children, in whom UIC is less than 50 μg/L. However, the iodine intake only slightly exceeds the recommended values, so median of UICs oscillates around the lower limit of references values.

## 

Goitre is the most frequent consequence of iodine deficiency, however, the insufficient supply of iodine leads not only to thyroid enlargement but also to a series of other disturbances, called – generally – iodine deficiency disorders [1]. School-age children are the population group most often assessed to reveal iodine deficiency, because they are the most efficient and practical group to survey, the group which usually reflects the status of the general population [2].

The best parameters to evaluate for this purpose in the population in question are goitre incidence and urine iodine concentration (UIC) [3].

Thyroid size has traditionally been determined by inspection and palpation, however thyroid ultrasonography provides a more precise and objective evaluation method. It is especially important in the cases of minor thyroid enlargement when the goitre assessment by palpation may be burdened with significant intra- and interobserver variability [4].

The previously proposed reference values for thyroid volume measured by ultrasonography (Delange F et al.) [5] were later re-evaluated by WHO (Zimmermann MB et al.) [6]. According to epidemiological criteria for assessing the iodine deficiency, based on the prevalence of goitre in school-age children, the value of 5% and above indicates iodine deficiency in such a degree which leads to thyroid enlargement [7].

Goitre incidence remains useful for initial assessment of the problem, however, this measure is generally not suitable for monitoring purposes. It is considered that estimation of UIC is a better way to describe the iodine deficiency in population. It is especially important if the goitre enlargement takes place but this enlargement is of a small degree and hence difficult to diagnose. The last issue makes also more difficult to assign goitre to proper grade of scale. In this situation, urine is tested for amount of iodine excretion, which allows assessing the seriousness of the iodine deficiency.

Urinary iodine concentration is a good marker of the recent dietary intake of iodine. It provides an adequate assessment of a population’s iodine nutrition and is now the index of choice for evaluating the degree of iodine deficiency, and for monitoring its correction [8]. According to epidemiological criteria for assessing iodine nutrition based on median UICs in school-age children, median below 100 μg/L indicates insufficient iodine intake. Optimal range is 100–199 μg/L.

According to the assumptions of declaration from year 1990, signatory countries (*i.a.* Poland) have obliged to eliminate the iodine deficiency in the coming years [9].

Elimination of iodine deficiency in Poland is based on iodine prophylaxis which was introduced in 1997 and is based on mandatory iodization of household salt with 20–40 mg KI/kg, supplementation of bottle fed infants with iodized formulas with 10.0 μg KI/100 mL, and a voluntary supplementation of pregnant and breast feeding women with additional 100–150 μg of iodine/day [10].

Based on multiple surveys it was found that the model of prophylaxis employed in Poland has proved to be effective. Thus, Poland became a country with sufficient iodine supplementation on the population level [11].

The prevention and control of iodine deficiency is a continuous process. It requires monitoring to be sustainable. There are many examples throughout the world where iodine deficiency has re-emerged as a public health problem, where once it was under control [12].

The aim of our study was to track the changes in iodine supply in recent years. School-age children were examined in 3 subsequent time points – in 1994, before establishment of currently operating model of iodine prophylaxis, in 1999 – 2 years after implementation of iodine prophylaxis and in 2010 – 14 years after its implementation.

## Subjects and methods

### Subjects

The subjects were healthy children living in Opoczno, the town in the centre of Poland (the Lodz Voivodship). The children were recruited from Primary School No 1. This study was part of the “Thyromobil action”, which was performed in several locations in Poland. The “ThyroMobil Van”, equipped with necessary testing devices and urine sample storage facilities, had usually been parked at the schoolyard.

104 children (54 girls and 50 boys; age range from 6 to 15 years), 207 children (104 girls and 103 boys; age range from 7 to 15 years), and 174 children (94 girls and 80 boys; age range from 8 to 15 years) were examined in 1994, 1999 and 2010, respectively.

Parents of all the children qualified for the experiment gave a written consent to the participation of their children in the study. The ethical committee approved the protocol (“ThyroMobil Project”).

## Methods

The height and the body mass of children were measured by using standard anthropometric techniques [13]. For the measurements, children took off their shoes and wore light indoor clothing.

The heights were recorded to the nearest millimeter, and the weights of children were recorded to the nearest 100 g. Body surface area (BSA) was calculated from the following formula: W^0. 425^ × H^0. 725^ × 71.84 × 10^- 4^, where: W – weight (kg); H – height (cm).

Thyroid palpation was performed, followed by ultrasound examination of the thyroid gland with a Siemens Sonoline SI-400 device (1994 and 1999) or a Siemens Sonoline Prima (2010) with a 7.5 MHz linear array transducer. Measurements were performed while the subjects were lying on the medical coach. The sum of lateral thyroid lobes volumes (determined sonographically) constituted the actual volume of the thyroid gland: the volume of the isthmus was skipped. The volume of thyroid lobe was calculated according to the following formula, proposed by Brunn et al. [14]: V(mL) = 0.479 × W × D × L, where: W – width (cm); D – depth (cm); lenght (cm).

The obtained data were compared to reference values for thyroid volume proposed by Zimmermann at al., adjusted for age and for body surface area (BSA) [6].

We also compared volume of the thyroid gland (V) to body surface area, calculating V/BSA ratio. In our opinion, this ratio (V/BSA) better reflects changes in thyroid volume in particular time points.

Urine samples were collected from each child, prior to the physical examination. In order to determine iodide concentration the modified catalytic method by Sandell and Kolthoff was used [15].

### Data and statistical analysies

The data were statistically analyzed, using non-parametric test for independent groups (Mann–Whitney Rank Sum test), Kruskal-Wallis One Way Analysis of Variance on Ranks, followed by Dunn’s test, Chi-Square Analysis and Pearson Correlation.

In all analyses, statistical significance has been considered achieved at a value of p<0.05.

Data processing, statistical analyses and figures were performed by using SigmaPlot 12.3 (Systat Software, Inc, San Jose, CA, USA) and Excel (Microsoft Corp., Redmond, WA, USA).

## Results

The mean values of age and of BSA in the examined schoolchildren were presented in Table 1.

**Table 1:** The number of examined children in gender groups, age, body surface area (BSA), urinary iodine concentration (UIC) and UIC distribution

**Data**	**all**				**UIC**		**UIC distribution**				
	**boys**	**girls**	**age ± SD**	**BSA**	**mean±SD**	**median (range)**	**<20**	**<50**	**<100**	**100-300**	**>300**
			**[y]**	**[m**^**2**^**]**	**[μg/L]**		**[%]**				
1994	88		9.74 ± 1.66	1.14 ± 0.20	54.6 ± 47.0	45.5	13.6	59.1	90.0	9.1	1.1
	42	46				(1.1 - 368.0)					
1999	207		9.69 ± 2.35	1.17 ± 0.26	110.7 ± 68.2	101.1	0	12.6	49.8	49.3	0.97
	103	104				(21.7 - 680.0)					
2010	170		11.97 ± 1.98	1.33 ± 0.23	110.6 ± 52.5	100.6	1.2	7.1	48.2	51.2	0.6
	80	90				(10.5 - 318.3)					

### Urine iodine concentration

The median of UICs was 45.5 μg/L (1994), 101.1 μg/L (1999) and 100.6 μg/L (2010).

**Figure 1:**
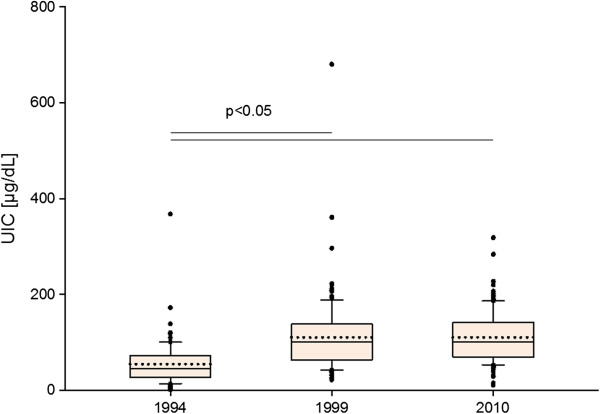
**Urinary iodine conentration (UIC) in the examined children.** Upper and lower limits of boxes are 75 and 25 percentile, respectively. Horizontal solid line and dotted line in the boxes are median and mean, respectively. Whiskers means standard deviation (SD). Points are scatter of UIC.

The differences between UIC were statistically significant between 1994 and 1999, and 1994 and 2010. The values of UICs in 1999 and 2010 did not differ (power of performed test with α = 0.05 is 1.000). The UIC values were presented in Figure 1 and Table 1.

**Figure 2:**
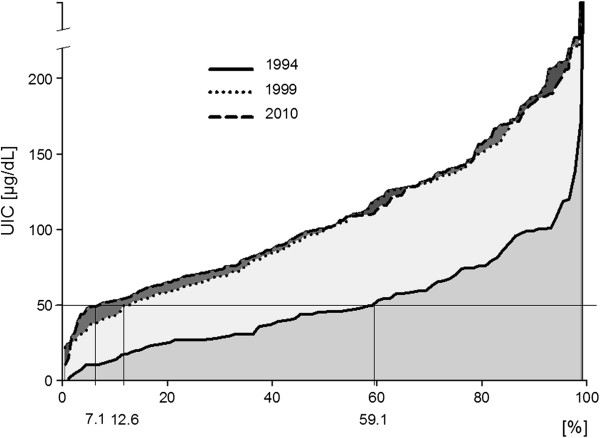
**Distribution of urinary iodine concentration (UIC).** Solid line, dotted line and the dash line are distribution of UIC in 1994, 1999 and 2010, respectively. The horizontal line is value of 50 μg/L of UIC.

Means of UICs between boys and girls were similar in 1994, 1999 and 2010 (differences were not statistically significant). The mean values of UIC in boys and girls were comparable in each time point. However, the distribution of iodine concentrations had changed (Figure 2).

Iodine concentrations below 50 μg/L were present in 59,1% children in 1994, in 12,6% children – in 1999 and in 7.1% children – in 2010.

### Goitre

**Figure 3:**
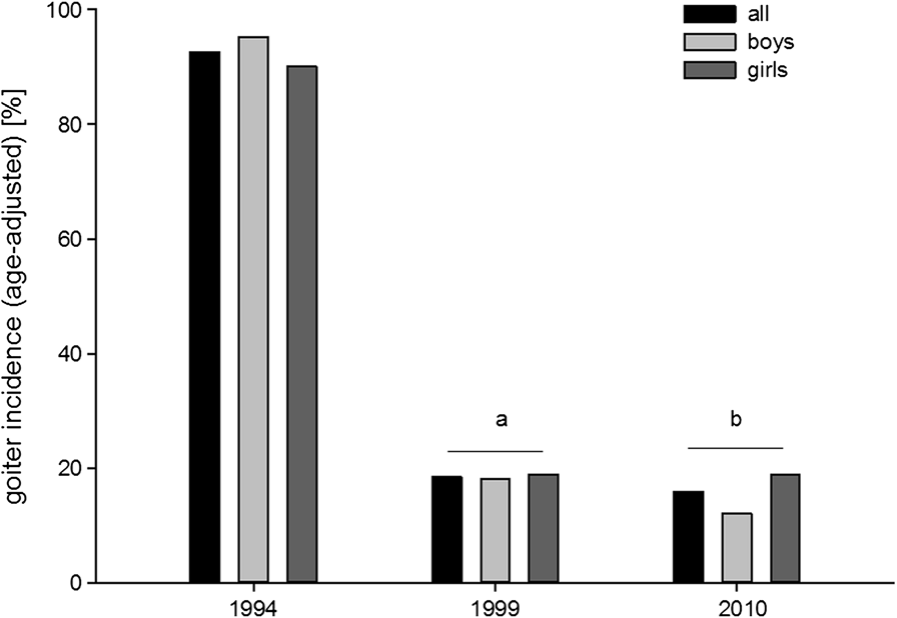
**Prevalence of goitre in examined children adjusted for age and gender.** a – p < 0.001 vs 1994; b – p < 0.001 vs 1994.

**Figure 4:**
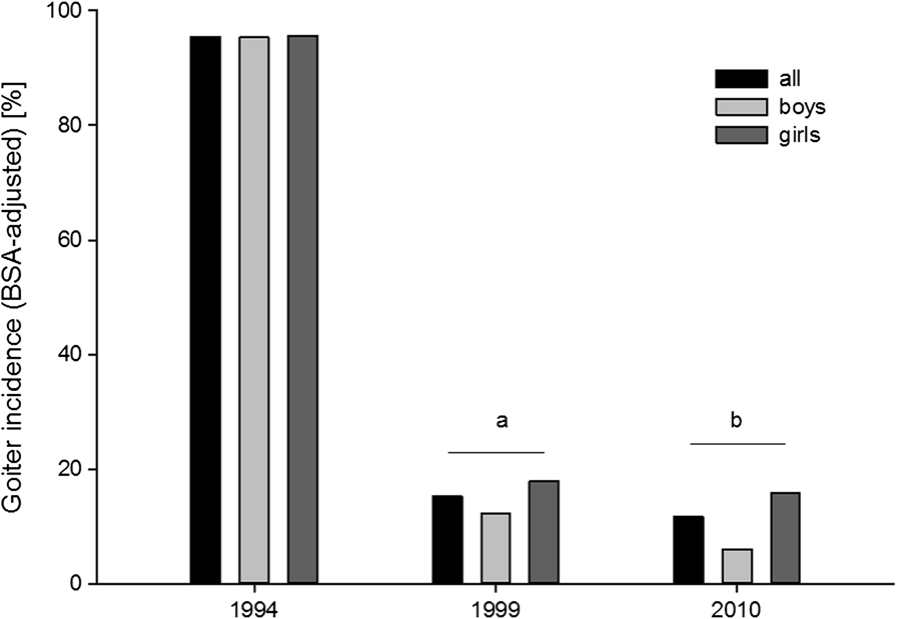
**Prevalence of goitre in examined children adjusted for body surface area (BSA) and gender.** a – p < 0.001 vs 1994; b – p < 0.001 vs 1994.

The values of goitre incidence – adjusted for age and for body surface area (BSA) – were presented in Figures 3 and 4, respectively.

The goitre prevalence adjusted for age was 92.6% (90.0% in girls and 95.1% in boys) in 1994; 18.5% (18.8% in girls and 18.1% in boys) in 1999 and 15.8% (18.8% in girls and 12.0% in boys) in 2010.

The goitre prevalence adjusted for body surface area (BSA) was 95.4% (95.6% in girls and 95.2% in boys) in 1994; 15.2% (17.8% in girls and 12.2 in boys) in 1999; 11.6% (15.7% in girls and 6.1% in boys) in 2010.

### V/BSA ratio

**Figure 5:**
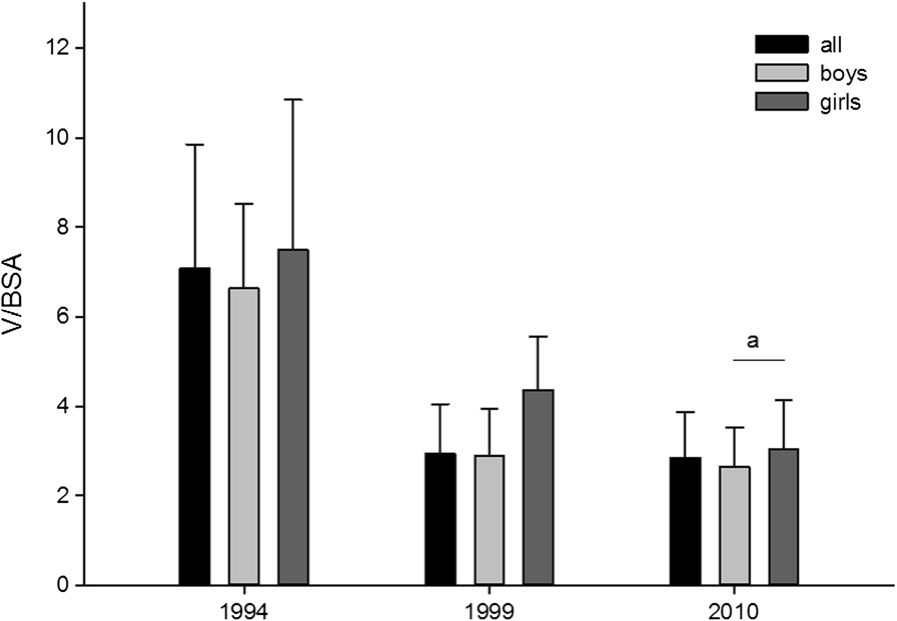
**Ratio of thyroid gland volume (mL) to body surface area (BSA) (m**^**2**^**) in examined children.** Whiskers means standard deviation (SD). a – p < 0.05 vs 1994; b – p < 0.05 vs 1994.

The values of ratio V/BSA (volume of thyroid gland to body surface area) were higher in 1994 (7.079 ± 2.775 in all, 6.628 ± 1.892 in boys; 7.491 ± 3.356 in girls) than in 1999 (2.935 ± 1.112 in all, 2.895 ± 1.049 in boys and 4.369 ± 1.175 in girls) (p<0.05) and in 2010 (2.846 ± 1.029 in all, 2.635 ± 0.892 in boys and 3.028 ± 1.107 in girls) (p<0.05; power of performed test with α = 0.05 is 1.000) (Figure 5).

**Figure 6:**
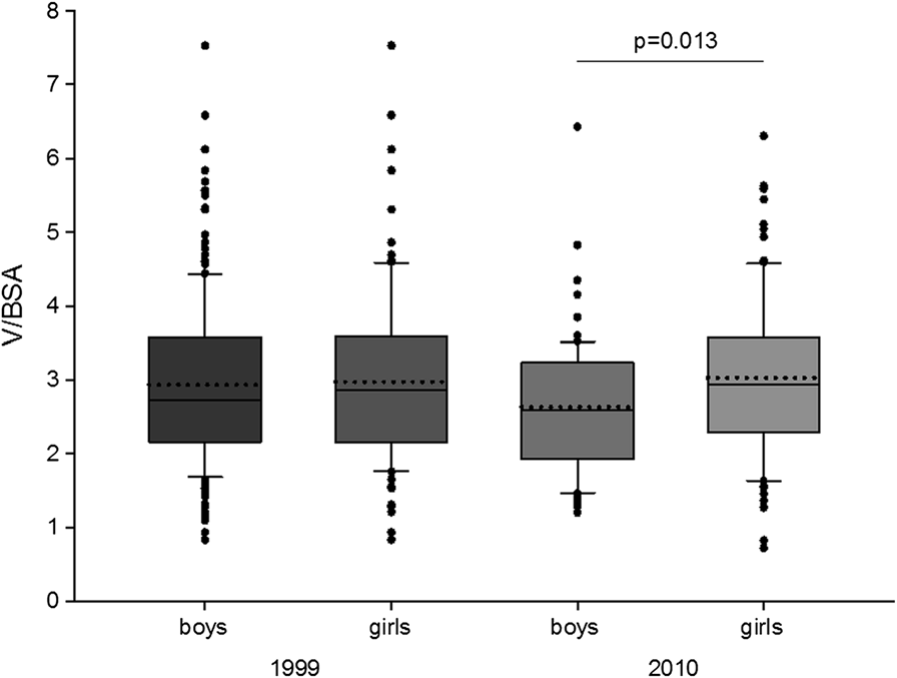
**Ratio of thyroid gland volume (mL) to body surface area (BSA) (m**^**2**^**) in examined children.** Upper and lower limits of boxes are 75 and 25 percentile, respectively. Horizontal solid line and dotted line in the boxes are median and mean, respectively. Whiskers means standard deviation (SD). Points are scatter.

The V/BSA value in girls was higher than in boys in 2010 (p = 0.013) (Figure 6).

## Discussion

The aim of the study was to demonstrate the effectiveness of iodine prophylaxis conducted in Poland. Results of the survey carried out in 1999 showed that iodine prophylaxis introduced in 1997 had proved to be effective [16,17].

A significant decrease in goitre incidence and thyroid size was observed, together with the increase in iodine excretion. The obtained results are consistent with other studies assessing the effectiveness of implemented iodine prophylaxis. Based on the studies from that period, Poland was classified as a country in which there was no iodine deficiency [18].

Eleven years later (2010), children from the same school were re-examined in order to follow the efficacy of conducted prophylaxis.

The value of mean and median of UICs has not changed, however this does not mean that there have been no changes. The analysis of UICs distribution shows that in 1999 the value scatter was much bigger than in 2010 (Figure 1). The percentage of children, in whom UIC was less than 50 μg/L decreased from 12.6 to 7.1% (Figure 2). Having analyzed the UICs distribution, it was found that the percentage of children with lower values of iodine concentration had decreased. Data demonstrate not only the effectiveness of iodine prophylaxis but also more even, homogenous iodine intake in comparison to year 1999. The greater uniformity of examined sample translates into easier grasping of statistical significances. The correlation between thyroid size and UICs value was observed only in 2010 (Pearson’s correlation coefficient was −0.165 with p=0.031). Such correlation was not seen in previous years. Moreover, in 2010 it was found that V/BSA ratio value was bigger in girls than in boys (p=0.013), which also indirectly points to greater uniformity of the examined population.

It is to be emphasized that the obtained values oscillate practically around lower optimal values. The optimal values of median of UICs in school-aged children are thought to be in the range between 100 and 200 μg/L.

The evaluation of UICs in school-aged children proved to be efficient reflection of iodine dietary intake. However, the values of median and mean are not the only parameters that reflect the iodine dietary intake. Analysis of data distribution, as well as analysis of percentage of children with iodine concentrations below normal values, is also very important.

It seems that the biggest problem is to define the reference values of thyroid volume, i.e. the values above which one can recognize thyroid enlargement – goitre. In 1999, when we were analyzing the obtained data, the reference values proposed by Delange et al. were in force – using these standards – the frequency of goitre was 37.5% (27.8% in girls, 48.0% in boys) in 1994, and 1.4% (1.0% in girls and 1.9% in boys) in 1999 [19]. Despite the fact that the reference values by Delange were considered to be too liberal, the degree of goitre severity, assessed according to them, correlated well with the degree of iodine deficiency assessed based on UIC.

In 2004, the reference values by Zimmermann et al. were presented and they were applied in our study. Having analyzed the obtained results, it was found that there had been a significant decrease in goitre incidence in 1999 and 2010 in comparison to 1994, taking into account both age and sex, as well as body surface area and sex. No differences in the goitre incidence between year 1999 and 2010 or between the group of girls and boys were found. However, the analysis of the obtained data may encounter common sense objections.

We are aware that before introduction of the iodine prophylaxis, there was an endemicity of goitre to moderate or average extent (20-40%); these data correlated with the iodine concentrations in urine samples. However, it is hard to believe that iodine deficiency was so large that it led to the goitre occurrence with the frequency of 90%. The evaluation of goitre incidence after the introduction of the iodine prophylaxis is also doubtful, as the frequency of goitre occurrence is still so high that it reaches the level of goitre endemicity (15-18%). This is in conflict with the values of UIC. We should expect the goitre incidence oscillating around the upper limit of normal values (ap. 5%). Therefore, one can assume that there are no reliable norms, to which the obtained results can be compared. Taking into consideration the fact that Delange’s and Zimmermann’s reference values were not the only ones – earlier quite rigorous reference values were proposed by Gutekunst et Martin-Teichert [20] – one can say that the reliable assessment of goitre incidence on the basis of currently available reference values is impossible. Polish reference values established by Szybinski et al. are also not representative as they were developed only with regard to age (not BSA); what is more, the study subjects were highly selected and did not reflect general pediatric population [21].

However, it does not mean, that the analysis of obtained results is useless. We have suggested comparing the thyroid volume measured by ultrasound to the child’s body surface area. The idea of such comparison is similar to the historic comparison of normal thyroid lobe to the examined person thumb [4].

The advantage of such comparison was the fact that the original data and not the interpreted ones were analyzed (thyroid enlarged vs. not enlarged). Moreover, data analyzed in such a manner are continuous data, which is especially important during childhood. In the case of the reference values, the rule of ranges enforces us to round the obtained results, which causes even more relativism. Furthermore, it is difficult to accept the reference values developed in population of children living far from Poland. When analyzing such results, one must be aware that in a particular site other goitrogenic factors might influence the population, the ethnic diversity should be also taken into account. All these issues make it very difficult to define universal reference values, which would be reliable.

The values of V/BSA ratio proves that after the implementation of iodine prophylaxis, the thyroid volume decreased significantly (Figure 5). Analysis of the obtained values between year 1999 and 2010 showed that V/BSA ratio value was higher in girls than in boys (especially high values were observed in girls aged 7–10).

Therefore, analysis of V/BSA ratio is very useful. It allows us not only to compare the thyroid volume in different time points but also in populations inhabiting different geographic regions. Thus, the differences in thyroid volume in children living in various geographic areas can be objectively determined, even in places classified as areas with sufficient iodine intake. In such cases, the influence of other factors, which might influence thyroid volume, apart from iodine intake, like ethnic factors, goitrogenic factors related to environmental pollution or consumption of specific diet, can be examined and considered.

## Conclusions

Iodine prophylaxis is a process which requires constant monitoring and adequate modification. Hitherto model of iodine prophylaxis has proved to be effective in eliminating the iodine deficiency.

Fifteen years after the implementation of iodine prophylaxis, the iodine intake is more even, homogenous, which translates into smaller scatter of UICs and less percentage of children, in whom UIC is less than 50 μg/L. However, the iodine intake only slightly exceeds the recommended values, so median of UICs oscillates around the lower limit of references values. Following the multidirectional approaches aimed at decreasing the salt intake, it may turn out in future that the iodine intake from the kitchen salt will decrease and it will be necessary to substitute this element in different way.

The comparison of thyroid volume to the BSA is a useful tool for controlling the impact of iodine intake on the thyroid volume. Values calculated by that means are the raw data, so the risk of data misinterpretation is minimized. At present, interpretation of obtained results based on existing ultrasound reference values poses a risk of misperception of the actual state.

## Abbreviations

UIC: Urine iodine concentration; BSA: Body surface area; V: Volume of the thyroid gland (measured by ultrasonography).

## Competing interests

The authors have no competing interests to declare.

## Authors’ contributions

ArZ – contributed to the design of the study and acquisition of data, its analysis and interpretation and prepared a draft of the manuscript. ZA, KWD, ACM, KKR – participated in examination of children involved in the study. AgZ – prepared a draft of the manuscript. MKL – revised the manuscript critically for important intellectual content. AL – contributed to the design, analysis and interpretation of data, and wrote the final version of the manuscript. All authors read and approved the final manuscript.
